# Are TSH normal reference ranges adequate for pregnant women?

**DOI:** 10.1590/2359-3997000000182

**Published:** 2016-08-01

**Authors:** Léa Maria Zanini Maciel

**Affiliations:** 1 Faculdade de Medicina de Ribeirão Preto Universidade de São Paulo Ribeirão Preto SP Brasil Divisão de Endocrinologia e Metabologia, Faculdade de Medicina de Ribeirão Preto, Universidade de São Paulo, Ribeirão Preto, SP, Brasil

According to some investigators, pregnancy represents a “stress test” for the thyroid gland (1) since an intact gland and an appropriate iodine supply are needed for an adequate hormone supply for mother and fetus. Patients with mild underlying thyroid disease or inadequate dietary iodine may fail the test and become hypothyroid. Typically, thyroid hormone production and daily iodine intake increase by approximately 50% in the first few weeks of pregnancy (2). This greater demand on the thyroid gland is due to a series of physiological changes occurring during pregnancy. One of the important alterations is the elevated level of human chorionic gonadotropin (hCG) which, by being structurally similar to TSH, has a direct stimulating action on the thyroid gland through the TSH receptor. During pregnancy, the hCG peak occurs at the end of the 1^st^ trimester, followed by a reduction to a plateau during the second and third trimesters. The thyrotropic effect of hCG results in an increased production of thyroid hormones (TH) which is reflected on a transitory increase in free T4 (FT4) at the end of the 1^st^ trimester (3,4). Concomitantly, the increase in hCG results in a reduction of TSH; with the progression of pregnancy and the decline of hCG there is an elevation of TSH (5). Compared to pre-pregnancy concentrations, thyroxine binding globulin (TBG) increases 2-3 times by about the 20^th^ week of gestation. This is due to the estrogen-stimulated increase in the hepatic production of TBG and to a reduced clearance of the sialylated, heavier, forms of the molecule. This elevation causes, on average, a 1.5-fold increase of total tri-iodothyronine l (TT3) and thyroxine (TT4) around the 16^th^ week of gestation. Extra TH production is also necessary to cover the losses due to placental deiodination.

During pregnancy, the maternal requirements of iodine increase due to several reasons (4,6). One of the mechanisms postulated is related to the greater renal loss of iodine, although its significance has been debated (7). At the same time, iodine is transported to the growing fetus through the placenta, being necessary in order to cover the increased maternal production of TH due to the increase in TBG.

The diagnosis of hypothyroidism during pregnancy is fully dependent on thyroid function tests. Four guidelines have been recently published by experts’ groups in North America, Europe and Brazil regarding the diagnosis and management of thyroid disease in pregnancy (1,6,8,9). For the interpretation of thyroid function tests, all guidelines similarly recommend 2.5 mUI/L as the upper normal limit for TSH in the 1^st^ trimester if the normal range has not been established for that region. All guidelines also warn about the use of FT4 in pregnancy. The ATA recommends the determination of TT4 and the calculation of the free T4 index (FTI) as preferable to FT4 by immunoassay, although some investigators have argued that this is a retrograde and misguided measure (10).

It is not a simple task to establish the normal range of thyroid function tests during pregnancy and some factors should be considered in the interpretation of such tests:

## A) PREANALYTICAL FACTORS

**Gestational age** – The changes in thyroid function tests according to gestational age have been elegantly shown in a study of 13,599 nulliparous women evaluated at 1 week intervals from the 6^th^ week to term. The study documented that TSH falls to minimal levels during the 10^th^ week, followed by a progressive increase until term, with its value in the 10^th^ week being half the value observed at the beginning of pregnancy. Studies on other populations (Finnish and Chinese) (11,12) have confirmed these results. Thus, a correct interpretation of thyroid function requires knowledge of gestational age.

**Presence of anti-thyroid antibodies** – Anti-peroxidase and anti-thyroglobulin antibodies are markers of thyroid autoimmunity. Consistent evidence has shown that women with increased levels of these antibodies tend to have higher TSH concentrations. These observations suggest that women with positive titers of these antibodies should not be included in studies of reference values for healthy controls, a rule that is often ignored.

**Iodine sufficiency** – Iodine is the essential substrate for TH synthesis. Considering the increased iodine requirements during pregnancy, it has been recommended that pregnant women ingest at least 250 µg/day of this element (7). Studies conducted on iodine-sufficient populations such as those of the United States, United Kingdom and the northeastern region of the state of São Paulo, Brazil, have revealed iodine deficiency in pregnant women (13-16). Iodine deficiency and excess can have different effects on thyroid function. It is recommended that reference populations used in studies of thyroid function in pregnancy are iodine replete. Ideally, this would be confirmed with urine iodine measurements.

**Multiple pregnancies** – Serum hCG concentrations tend to be higher and TSH concentrations tend to be lower in women with multiple pregnancies (17). The practical implication of these observations is that TSH concentration may be lower in women carrying twins or in multiparous women and that these women should be excluded from the determination of population reference ranges.

**Ethnicity** – Several studies have demonstrated differences in TH concentrations among ethnic groups (18), and some have suggested that Black women have lower TSH values, while Asian women have higher TSH values compared to Caucasian women.

**Time of collection** – Circadian TSH rhythm has been observed in pregnant women (19) as well as in non-pregnant women, with this circadian variation persisting in the 2^nd^ and 3^rd^ trimesters. Thus, failure to standardize collection time may interfere with the results and interpretation of the tests.

## B) ANALYTICAL FACTORS

**Thyroid hormones** – In addition to representing a stress test for the maternal thyroid, pregnancy also represents a stress test for laboratory immunoassays, which may be affected by the changes in carrier proteins that occur during pregnancy, participating in TBG, and by the increase in free fatty acids and the reduction of albumin. While some investigators have detected good agreement between immunoassays, others have not (20-22). All guidelines recommend caution in the interpretation of FT4 data during pregnancy and the constant use of the reference values established by the laboratory, when available.

**TSH** – Different immunoassays result in different TSH values, as shown in Table 1. In general, the 97.5^th^ percentile of TSH for the 1^st^ trimester is located in two groups: according to the Architect, Beckman and Immulite platform, it is about 3.0 mIU/L, while according to Centaur and Roche it s close to 4 mIU/L It should be pointed out that only 4 of 27 studies, 2 of them using the Abbott Architect platform (23,24), one using te Immulite platform (25) and one the Centaur platform (2), showed values close to or below the cut-off of 2.5 mIU/L.

**Table 1:** Summary of pregnancy thyroid function test reference interval using different methods

TSH mIU/L							

Trimester:		First		Second		Third	

Group	Studies	2.5^**th**^	97.5^**th**^	2.5^**th**^	97.5^**th**^	2.5^**th**^	97.5^**th**^
Architect	8	0.13	3.00	0.22	3.20	0.37	3.49
Beckman	4	0.12	3.12	0.29	3.51	0.25	3.93
Centaur	6	0.08	3.55	0.05	4.50	0.47	4.54
Immulite	5	0.09	3.09	0.30	3.21		
Roche	7	0.15	4.00	0.31	4.17	0.38	4.15

Source: McNeil AR, Stanford PE (28).

Each number is the mean value for all studies for a given method group.

The objective of the article by Rosario and cols. (26) published in this volume of *Archives of Endocrinology and Metabolism*, was to establish the normal TSH range for the 1^st^ trimester of pregnancy and to correlate the obstetric and neonatal outcome with the maternal serum TSH concentrations.

The study obeyed the criteria recommended by the NACB (27) for the establishment of reference values, although approximately 47% of the pregnant women were multiparous. While the authors stated that there is no difference in TSH values between primigestae and multiparous women, they did not mention iodine sufficiency in these pregnant women, as was also the case for other studies conducted with the same objective. Using the Immulite 2000 platform, it was observed that the 97.5^th^ percentile of the values obtained for the sample was 2.68 mIU/L at a median gestational age of 9 weeks, a value close to that recommended by published guidelines.

What is the importance of establishing the normal range for pregnant women? It is the fact that the use of the range for non-pregnant women may possibly lead to an erroneous “normal” diagnosis for a pregnant woman with high TSH and subclinical hypothyroidism and lead to the consideration of hyperthyroidism in a normal pregnant woman due to the low TSH values observed in the 1^st^ trimester of pregnancy, as was the case for 19.4% of the pregnant women studied in this investigation. Also, the use of reference values based on studies of other populations with different backgrounds may introduce a bias in the assessment of the local cohort.

Thus, we conclude that these results are highly relevant for professionals who work in the same region and who use the same laboratory system of hormone determination.
